# IL-15 Promotes Polyfunctional NK Cell Responses to Influenza by Boosting IL-12 Production

**DOI:** 10.4049/jimmunol.1701614

**Published:** 2018-02-28

**Authors:** Helen R. Wagstaffe, Carolyn M. Nielsen, Eleanor M. Riley, Martin R. Goodier

**Affiliations:** * Department of Immunology and Infection, London School of Hygiene and Tropical Medicine, London WC1E 7HT, United Kingdom;; † Jenner Institute, University of Oxford, Oxford OX3 7DQ, United Kingdom; and; ‡ The Roslin Institute and Royal (Dick) School of Veterinary Studies, University of Edinburgh, Easter Bush, Midlothian EH25 9RG, United Kingdom

## Abstract

IL-15 is a key regulator of NK cell maintenance and proliferation and synergizes with other myeloid cell–derived cytokines to enhance NK cell effector function. At low concentrations, *trans*-presentation of IL-15 by dendritic cells can activate NK cells, whereas at higher concentrations it can act directly on NK cells, independently of accessory cells. In this study, we investigate the potential for IL-15 to boost responses to influenza virus by promoting accessory cell function. We find that coculture of human PBMCs with inactivated whole influenza virus (A/Victoria/361/2011) in the presence of very low concentrations of IL-15 results in increased production of myeloid cell–derived cytokines, including IL-12, IFN-α2, GM-CSF, and IL-1β, and an increased frequency of polyfunctional NK cells (defined by the expression of two or more of CD107a, IFN-γ, and CD25). Neutralization experiments demonstrate that IL-15–mediated enhancement of NK cell responses is primarily dependent on IL-12 and partially dependent on IFN-αβR1 signaling. Critically, IL-15 boosted the production of IL-12 in influenza-stimulated blood myeloid dendritic cells. IL-15 costimulation also restored the ability of less-differentiated NK cells from human CMV-seropositive individuals to respond to influenza virus. These data suggest that very low concentrations of IL-15 play an important role in boosting accessory cell function to support NK cell effector functions.

## Introduction

Interleukin-15 is essential for the survival, proliferation, and functional integrity of NK cells and is being exploited to enhance NK cell–mediated immunotherapies (1, 2). IL-15 augments NK cell expression of perforin, granzyme B, natural cytotoxicity receptors NKp30 and NKp44 (3), and the activating receptor NKG2D (4). The potency of IL-15, even at very low concentrations, is due, in part, to its presence as a complex with the α-chain of its own receptor (IL-15Rα) at the surface of APCs (5), where it can be presented to the same cell (*cis*-presentation) or to neighboring cells (*trans*-presentation), such as NK cells and CD8^+^ T cells that express IL-15Rβ and the common γ-chain receptor (IL-15Rβγc) (6).

Dendritic cells (DCs) can be induced to present IL-15 at their surface by microbial ligands signaling through TLR and by innate cytokines, such as GM-CSF and type I IFN (7, 8). DC-mediated NK cell activation is dependent, in part, on IL-15 that polarizes to the DC–NK cell synapse during conjugate formation (9). Although IL-15 is believed to mediate the majority of its effects via *trans*-presentation by IL-15Rα, at high concentrations it can bind directly to IL-15Rβγc and, thereby, activate NK cells (10). Furthermore, at very high concentrations, free IL-15 may bind to IL-15Rα on neighboring cells for *cis*- or *trans*-presentation (5, 11). Low concentrations of IL-15 alone induce negligible NK cell activation, but IL-15 is highly synergistic with other cytokines and with recall Ags, such as influenza, for NK cell CD25 and IFN-γ expression (12, 13).

IL-15 *trans*-presentation is being explored for cancer immunotherapy; induction of constitutive expression of IL-15 and IL-15Rα by DCs or the use of soluble IL-15/IL-15Rα complexes has been shown to enhance NK cell antitumor activity in vitro and in preclinical mouse studies (reviewed in Ref. 14). Another strategy for NK cell immunotherapy includes activation of NK cells with IL-12, IL-15, and IL-18 prior to adoptive transfer, which reduces tumor growth in mice (15). Preactivation of PBMCs with high concentrations of IL-15 can also restore impaired NK cell cytotoxicity of SIV-infected macaques (16). Although these studies are consistent with direct NK cell priming by IL-15 at high concentration, or synergy with myeloid cell–derived cytokines at low concentration, the potential for IL-15 to amplify the myeloid cell response has not been thoroughly explored. However, one study has shown that TLR-induced maturation of DCs is enhanced in the presence of IL-15, leading to increased NK cell cytotoxicity toward human papillomavirus (HPV)-infected cells (17).

We hypothesized that, in addition to NK cell activation by *trans*-presentation and other direct effects, IL-15 could have indirect effects on the response of human NK cells to viruses by promoting NK cell–activating cytokines from accessory cells. We show that very low concentrations of IL-15 (0.75 ng/ml) dramatically enhance the production of IL-12, IFN-α, IL-1β, and GM-CSF from myeloid accessory cells in response to inactivated whole influenza virus (A/Victoria/361/2011) (H3N2) and that increased production of IFN-α and, in particular, IL-12 is associated with heightened and sustained activation of NK cells and polyfunctionality of the responding NK cells. Furthermore, IL-15–mediated enhancement preferentially boosts IL-12 production in myeloid DCs (mDCs) compared with other blood DC populations and monocytes. These studies suggest that IL-15–enhanced accessory cell function may potentiate NK cell responses, providing an additional avenue of interest for boosting NK cell effector responses in vaccination and NK cell–mediated immunotherapy.

## Materials and Methods

### Study participants

Volunteers were recruited from the staff and students at the London School of Hygiene and Tropical Medicine (*n* = 84) using an anonymized volunteer database. The study was approved by the London School of Hygiene and Tropical Medicine Research Ethics Committee (reference numbers 6237 and 6324). Human CMV (HCMV) serostatus was determined for each donor with HCMV IgG ELISA (Biokit, Barcelona, Spain) using plasma collected from heparinized whole blood. Donors ranged in age from 20 to 77 y, with a median age of 32 y. Twenty-nine (35%) of the donors were male, and 48% were HCMV seropositive.

### PBMC isolation and in vitro culture assays

PBMCs were isolated from heparinized whole blood using Histopaque 1077 (Sigma-Aldrich, Gillingham, U.K.) gradient centrifugation. Cells were rested for 2 h and were used fresh (blocking experiments and IL-12 intracellular staining) or were cryopreserved in liquid nitrogen (all other experiments). Before use, frozen cells were thawed and washed in RPMI 1640 supplemented with 100 U/ml penicillin/streptomycin and 20 mM l-glutamine (Life Technologies, Thermo Fisher). Cells were counted using a Countess II FL Automated Cell Counter (Invitrogen, Thermo Fisher); average viability after thaw was 86%. A total of 3 × 10^5^ cells per well was cultured in RPMI 1640 supplemented as above with 5% pooled human AB serum for 6, 9, or 18 h at 37°C in 96-well round-bottom plates with 2 μg/ml H3N2 (IVR-165; National Institute for Biological Standards and Control, Potters Bar, U.K.), with or without 0.75 ng/ml recombinant human IL-15 (PeproTech, London, U.K.). Concentrations were determined by prior titration; 2 μg/ml H3N2 was the lowest concentration to induce significant NK cell IFN-γ upregulation without the presence of additional cytokines, and 0.75 ng/ml IL-15 was previously shown to be the lowest concentration to synergize with other cytokines for NK cell activation, without significant NK cell activation alone (13). Additional cultures were stimulated with a high concentration of cytokines consisting of IL-12 (5 ng/ml; PeproTech) and IL-18 (50 ng/ml; R&D Systems, Oxford U.K.). The following Abs were used for blocking experiments: anti–IL-2 (3 μg/ml; BD Biosciences, Oxford, U.K.), rat IgG2a isotype control (3 μg/ml; eBioscience, Thermo Fisher), anti–IL-12 (3 μg/ml; BD Biosciences), anti–IFN-αβR2 (1 μg/ml; Merck Millipore, Watford, U.K.), and combined mouse IgG1 and IgG2a isotype controls (3 μg/ml final; eBioscience). GolgiStop (monensin; 1/1500 concentration) and GolgiPlug (brefeldin A; 1/1000 final concentration; both from BD Biosciences) were added for the final 3 h of culture. Culture supernatants were collected and stored at −80°C. For control experiments, NK cells were purified (mean purity 87%) using an NK Cell Isolation Kit (Miltenyi Biotec), and 2 × 10^5^ cells were stimulated for 18 h under the conditions described above for PBMC cultures. Cells were stained with NK cell activation markers as before. For IL-12 intracellular staining experiments, 2 × 10^6^ cells per well were cultured as above for 18 h, with GolgiStop and GolgiPlug for the final 5 h.

### Flow cytometry and Luminex

Cells were stained in 96-well round-bottom plates for surface markers, including viability marker (Fixable Viability Dye eFluor 780; eBioscience) in FACS buffer (PBS containing 0.5% FCS, 0.05% sodium azide, and 2 mM EDTA) for 30 min after blocking Fc receptors for 5 min with Fc Receptor Blocking Reagent (Miltenyi Biotec). Cells were then washed in FACS buffer and fixed and permeabilized using a BD Cytofix/Cytoperm Kit, according to the manufacturer’s instructions. Cells were then stained for intracellular markers with FcR blocking for 15 min and washed again; finally, cells were resuspended in 300 μl of FACS buffer and transferred to alpha tubes for acquisition on a BD LSR II flow cytometer. The following fluorophore-labeled Abs were used: anti-CD3–V500 (clone UCHT1), anti-CD56–PE–Cy7 (clone NCAM16.2), anti-CD107a–FITC (clone H4A3), anti-HLA-DR–PE (clone TU36) (all from BD Biosciences), anti-IFN-γ–allophycocyanin (clone 45.B3), anti-CD86–Alexa Fluor 488 (clone IT2.2), anti-CD11c–PerCP-Cy5.5 (clone 3.1), anti-CD16–PE/Dazzle (clone 3G8), anti-CD14–Alexa Fluor 700 (clone 63D3) (all from BioLegend, London, U.K.), anti-CD25–PerCP-Cy5.5 (clone BC96), anti-CD57–eFluor 450 (clone TB01), anti-CD19–PE–Cy5 (clone HIB19), anti-CD123–eFluor 450 (clone 6H6), and anti–IL-12(p40)–eFluor 660 (clone C17.8) (all from eBioscience). Cells were acquired using FACSDiva software, and data were analyzed using FlowJo v10 (TreeStar, Ashland, OR). FACS gates were set using unstimulated cells or fluorescence minus one controls, and samples with <100 NK cell events were excluded. Concentrations of GM-CSF, IFN-α2, IFN-γ, TNF-α, IP-10, IL-1β, IL-10, and IL-12p70 in cell culture supernatants were determined using Luminex technology (Merck Millipore) and Bio-Plex software (Bio-Rad, Watford, U.K.).

### Statistics

Statistical analysis was performed using GraphPad Prism version 7.01 (GraphPad, La Jolla, CA). Functional responses were compared using the Wilcoxon signed-rank test, and intergroup comparisons between HCMV-seropositive and HCMV-seronegative individuals were performed using the Mann–Whitney *U* test. Correlation analysis was performed using linear regression. Significance levels are assigned as *p < 0.05, **p < 0.01, ***p < 0.001, and ****p < 0.0001 for all tests. Analysis and presentation of polyfunctional NK cell data were performed using SPICE version 5.1 (downloaded from https://niaid.github.io/spice/).

## Results

### Nanogram concentrations of IL-15 boost and sustain functional NK cell responses to H3N2

**FIGURE 1.**
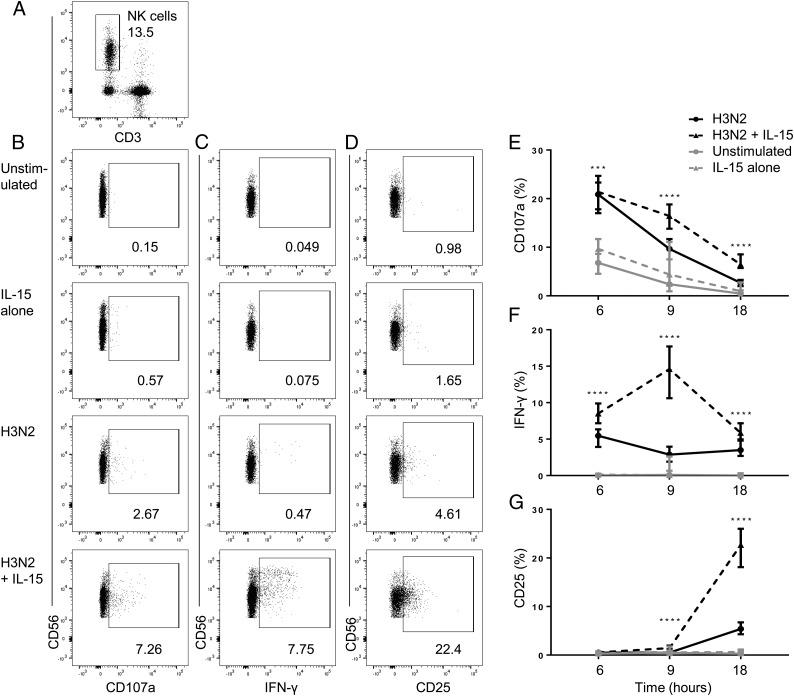
Nanogram concentrations of IL-15 boost and sustain functional NK cell responses to H3N2. PBMCs were cultured in vitro for 6, 9, and 18 h in medium alone (unstimulated; *n* = 12), IL-15 alone (*n* = 12), H3N2 (*n* = 62), or H3N2 plus IL-15 (*n* = 62). (**A**) NK cells are gated as CD56^+^CD3^−^ lymphocytes. Flow cytometry gating strategy for NK cell CD107a (**B**), IFN-γ (**C**), and CD25 (**D**) responses after an 18-h in vitro culture in one representative individual. Numbers shown are the percentage of total NK cells positive for each marker. Graphs show the percentage of the total NK cell population expressing CD107a (**E**), IFN-γ (**F**), or CD25 (**G**). Data are median and 95% confidence interval. ***p < 0.001, ****p < 0.0001 H3N2 alone versus H3N2 plus IL-15 by the Wilcoxon signed-rank test.

To determine the effect of a low concentration of exogenous IL-15 on the frequency and kinetics of NK cell responses to influenza, IFN-γ, CD107a, and CD25 were measured at 6, 9, and 18 h after stimulation of human PBMCs with H3N2 in the presence or absence of 0.75 ng/ml recombinant human IL-15. The flow cytometry gating strategy is shown in Fig. 1A–D. H3N2 alone induced high frequencies of NK cells expressing each of the three activation markers, but there was no significant response to IL-15 alone. At all time points, the percentage of expression of all markers was significantly higher when cells were cultured with H3N2 plus IL-15 compared with cells cultured with H3N2 alone (Fig. 1E–G), with each marker displaying distinctly different kinetics (frequencies of cells expressing CD107a, IFN-γ, and CD25 peaking at 6, 9, and 18 h, respectively). A low frequency of NK cells showed spontaneous degranulation (CD107a expression) at early time points (Fig. 1E), whereas there was little or no IFN-γ production or CD25 induction in unstimulated cultures (Fig. 1F, 1G). Increased responses to H3N2 in the presence of IL-15 were reflected in increased mean fluorescence intensities (MFIs) for all NK cell functional markers at their optimal time points. A small, but significant, increase was observed in CD107a MFI at 6 h (H3N2 median 747 units, interquartile range [IQR] 718–776; H3N2+IL-15 median 768 units, IQR 735–813, *p* ≤ 0.0001), IFN-γ MFI increased substantially by 9 h (H3N2 median 681 units, IQR 523–814; H3N2+IL-15 median 1037 units, IQR 864–1179, *p* ≤ 0.0001), and a significant shift in CD25 MFI was observed by 18 h (H3N2 median 72.5 units, IQR 34.7–153.8; H3N2+IL-15 median 371.5 units, IQR 249–508, *p* ≤ 0.0001). Thus, very low concentrations of IL-15 boost the NK cell response to H3N2 stimulation and lead to sustained CD107a and IFN-γ production compared with untreated cultures.

NK cells are a heterogeneous population of cells; CD56^bright^ subsets are highly responsive to cytokines, whereas the more mature CD56^dim^CD57^+^ subset is known to be less responsive to cytokines but maintains cytotoxic function (18). Therefore, we analyzed the response of each of these subsets to IL-15 (flow cytometry gating strategy shown in Supplemental Fig. 1A). As expected, the early degranulation response was highest in the most mature (CD56^dim^CD57^+^) subset and IFN-γ production was highest in the less mature (CD56^dim^CD57^−^) subsets at the peak of each response (Supplemental Fig. 1B, 1F). However, at 6 h, IL-15 had little effect on degranulation in the CD56^dim^CD57^+^ NK cell population, whereas, by 9 h, the heightened response in the presence of IL-15 was pronounced within the CD56^bright^ and CD56^dim^ subsets (Supplemental Fig. 1B, 1E). Enhancement of NK cell IFN-γ and CD25 responses to influenza by low concentrations of IL-15 was evident to a similar extent in all NK cell subsets (Supplemental Fig. 1C–J).

### IL-15 enhances NK cell polyfunctionality

**FIGURE 2.**
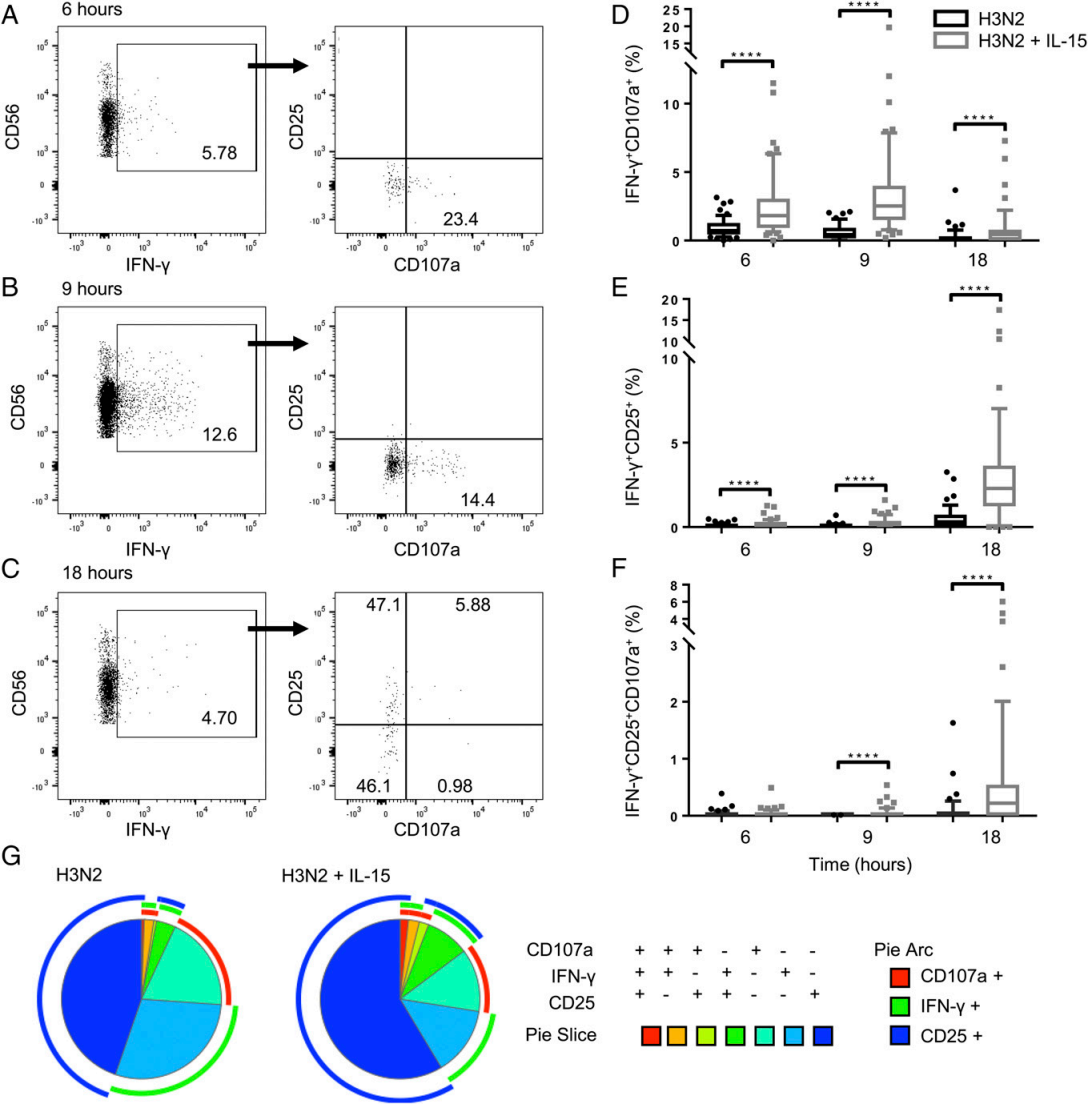
IL-15 enhances NK cell polyfunctionality. CD107a and CD25 expression of IFN-γ–secreting NK cells was gated at 6 h (**A**), 9 h (**B**), or 18 h (**C**). Plots show NK cell function in one representative donor; number is the percentage of the parent population after stimulation with H3N2 plus IL-15. Frequencies of IFN-γ^+^CD107a^+^ double-positive (**D**), IFN-γ^+^CD25^+^ double-positive (**E**), and IFN-γ^+^CD25^+^CD107a^+^ triple-positive (**F**) NK cells, after Ag stimulation with IL-15 (gray) or without IL-15 (black), at each time point (*n* = 62). Graphs are box-and-whisker plots with 10–90th percentiles. (**G**) Median distribution of triple-, double-, and single-positive NK cells after stimulation with H3N2 alone or H3N2 with IL-15 for 18 h are shown as pie charts as the percentage of total NK cells (pie slice) and the proportion expressing each marker (pie arc). ****p < 0.0001, Wilcoxon signed-rank test.

Because IL-15 appeared to enhance all three NK cell functions to a similar degree, we considered the possibility that this was due to a fraction of NK cells being highly sensitive to the effects of IL-15 and responding in a polyfunctional manner. We examined coexpression of IFN-γ with CD107a, CD25, or both at each time point (flow cytometry gating strategy shown in Fig. 2A–C). No polyfunctional NK cells were detected in unstimulated cultures or in cultures containing only IL-15 (Supplemental Fig. 2A–C). When cells were cultured with H3N2 alone, very few double- or triple-positive NK cells were detectable (Fig. 2D–F); however, after 6 or 9 h of culture with H3N2 plus IL-15, a considerable population of NK cells was double positive for IFN-γ and CD107a (Fig. 2D); by 18 h, cells coexpressing IFN-γ and CD25 (Fig. 2E), together with a small, but statistically significant, population of triple-positive NK cells (Fig. 2F), were detectable. Importantly, influenza virus stimulation in the presence of as little as 0.75 ng/ml IL-15 was almost as effective at inducing polyfunctional NK cells as much higher concentrations of a combination of IL-12 and IL-18 (5 and 50 ng/ml, respectively, Supplemental Fig. 2A–C). Polyfunctionality of NK cells was also evident from the statistically significant correlation between expression of the different functional markers by NK cells cultured with H3N2 plus IL-15 compared with those cultured with H3N2 alone (Supplemental Fig. 2D–I). Overall, therefore, IL-15 significantly increased the frequency of polyfunctional NK cells responding to influenza virus.

### IL-15 enhances in vitro production of myeloid cell–derived cytokines

**FIGURE 3.**
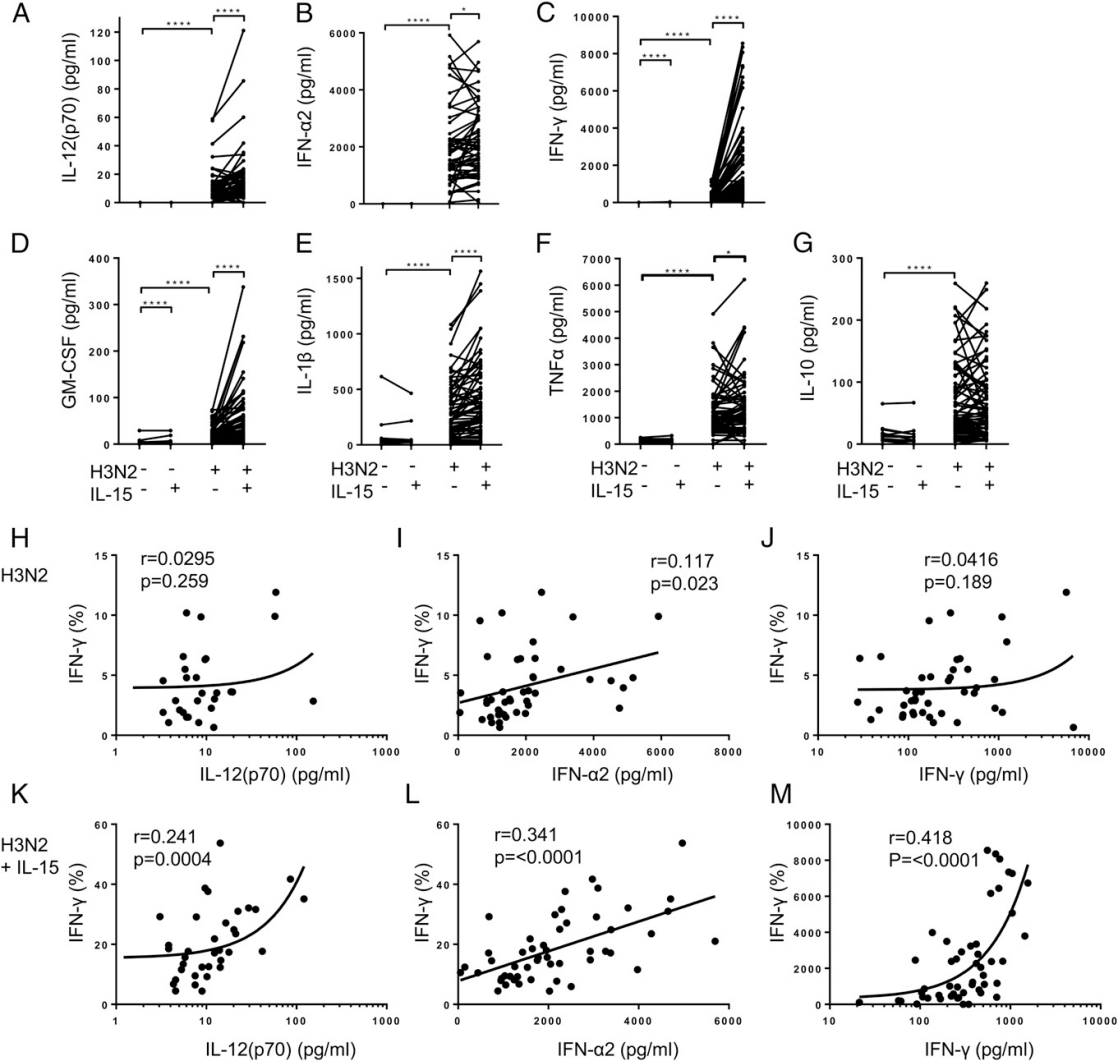
IL-15 enhances in vitro production of myeloid cell–derived cytokines. Supernatants were collected from whole PBMCs cultured with medium (*n* = 20), IL-15 (*n* = 20), H3N2 (*n* = 73), or H3N2 with IL-15 (*n* = 73) for 18 h, and concentrations of IL-12p70 (**A**), IFN-α2 (**B**), IFN-γ (**C**), GM-CSF (**D**), IL-1β (**E**), TNF-α (**F**), and IL-10 (**G**) were determined by Luminex technology. The correlation between NK cell IFN-γ production was determined by intracellular cytokine staining, and the concentration of IL-12 (p70) (**H** and **K**), IFN-α2 (**I** and **L**), and IFN-γ (**J** and **M**) was determined by Luminex after stimulation with H3N2 (H–J) or H3N2 with IL-15 (K–M). (A–G) Graphs are before-and-after plots. *p < 0.05, ****p < 0.0001, Wilcoxon signed-rank test. (H–M) Correlations were measured by linear regression, with statistical significance determined as a *p* value < 0.05.

Because NK cells respond to cytokines released from activated accessory cells, such as DCs and monocytes, supernatants were collected from cultures at 18 h, and cytokine concentrations were measured by Luminex. H3N2 alone induced secretion of significant concentrations of IL-12 (p70), IFN-α2, IFN-γ, GM-CSF, IL-1β, TNF-α, and IL-10 (Fig. 3A–G). However, addition of low concentrations of IL-15 to H3N2 resulted in further significant increases in the secretion of all of these cytokines, with the exception of IL-10 (Fig. 3G). IL-15–mediated enhancement of IL-12 secretion was observed in 42 of 73 donors (57.5%), with an average 2.1-fold increase in these individuals (Fig. 3A). IL-15 costimulation increased IFN-α2 secretion in 66% of individuals compared with H3N2 alone, with a median 1.2-fold increase (Fig. 3B), as well as enhanced IFN-γ by an average of 5.6-fold (Fig. 3C), consistent with IL-15 boosting of NK cell IFN-γ responses (Fig. 1). In contrast, IL-15 alone induced only modest increases in IFN-γ and GM-CSF production (median increase of only 8.18 pg/ml for IFN-γ and an increase in GM-CSF in only 3 of 20 donors) compared with unstimulated cells (Fig. 3C, 3D), and IL-15 alone had no effect on IL-12 (p70), IFN-α2, IL-1β, TNF-α, or IL-10 secretion (Fig. 3A, 3B, 3E–G).

In the presence of H3N2 and IL-15, the percentage of NK cells producing IFN-γ at 18 h was significantly correlated with the secreted concentrations of IL-12 and type I IFN (Fig. 3H–M). However, there was no enhanced correlation between NK cells producing IFN-γ and GM-CSF, TNF-α, or IL-1β with IL-15 (GM-CSF: *r* = +0.385, *r* = +0.391 with IL-15; TNF-α: *r* = +0.231, *r* = +0.285 with IL-15; IL-1β: *r* = +0.197, *r* = +0.331 with IL-15), suggesting that IL-12 and/or IFN-α2 might be driving the enhanced NK cell IFN-γ response. In summary, IL-15 dramatically enhances the secretion of myeloid cell–derived cytokines, and the secretion of two of these cytokines (IL-12 and IFN-α2) is strongly correlated with NK cell function.

### IL-12 is critical for IL-15–mediated enhancement of NK cell responses and generation of polyfunctional NK cells

**FIGURE 4.**
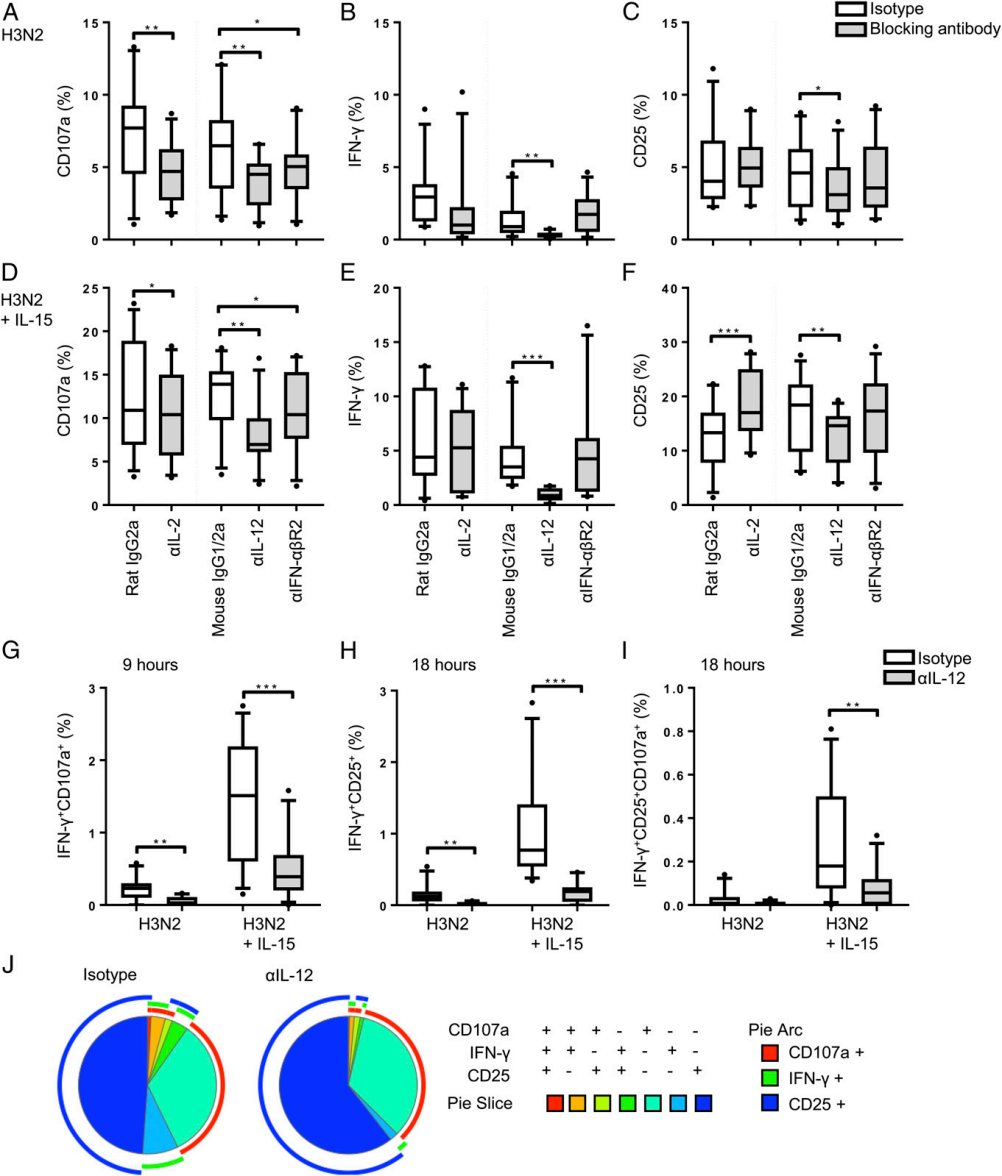
IL-12 is critical for IL-15–mediated enhancement of NK cell responses and generation of polyfunctional NK cells. PBMCs were cultured for 6, 9, and 18 h with H3N2 (**A**–**C**) and H3N2 plus IL-15 (**D**–**F**) in the presence of IL-12–, IFN-αβR2–, or IL-2–blocking Ab or the appropriate isotype control. Graphs show CD107a (A and D), IFN-γ (B and E), and CD25 (C and F) responses after an 18-h culture, and frequencies of IFN-γ^+^CD107a^+^ (**G**), IFN-γ^+^CD25^+^ (**H**) double-positive and IFN-γ^+^CD25^+^CD107a^+^ triple-positive (**I**) NK cells after 9 h (peak of IFN-γ response) or 18 h (peak of CD25 response) (*n* = 11). Graphs are box-and-whisker plots with 10–90th percentile. (**J**) Median distributions of triple-, double-, and single-positive NK cells after stimulation with H3N2 and IL-15 for 18 h are shown as the percentage of total NK cells (pie slice) and the proportion expressing each marker (pie arc). *p < 0.05, **p < 0.01, ***p < 0.001, Wilcoxon signed-rank test.

Because IL-12 and IFN-α2 were induced by coculturing PBMCs with H3N2 and IL-15, and because enhanced NK cell function was correlated with the concentrations of these two monokines, we tested the hypothesis that the effects of IL-15 on NK cells were mediated via IL-12 and/or type I IFNs. Neutralizing Abs against IL-12, IFN-αβR2, and IL-2 were added to cultures stimulated with H3N2 (with and without IL-15) for 6, 9, and 18 h. All three NK cell functional responses were reduced in the presence of blocking Ab to IL-12 by 18 h (Fig. 4A–F), as well as at the earlier time point of 9 h (data not shown); in particular, the frequency of CD107a- or IFN-γ–producing cells induced by H3N2 plus IL-15 was reduced to that observed in the absence of IL-15 (Fig. 4A–E). Degranulation was also dependent, in part, on type I IFNs, because blockade of the IFN-αβR2 receptor led to a partial reduction in CD107a expression in cultures treated or not with IL-15 (Fig. 4A, 4D). Blocking IL-2 also reduced CD107a upregulation after H3N2 stimulation, irrespective of the presence or absence of IL-15 (Fig. 4A, 4D). However, although anti–IL-2 reduced the NK cell IFN-γ response to H3N2, no such reduction was seen in the response to H3N2 plus IL-15, suggesting that IL-15 may be substituting for IL-2 in this assay.

In addition to reducing the overall frequencies of CD107a^+^, IFN-γ^+^, and CD25^+^ NK cells, IL-12 blockade had a marked effect on the induction of polyfunctional NK cells. IL-12 neutralization reduced the frequencies of double- and triple-positive NK cells to the levels observed without IL-15 (Fig. 4G–J). These data suggest that the heightened NK cell response to H3N2 and the generation of polyfunctional NK cells in response to IL-15 are dependent on accessory cell IL-12. Furthermore, correlations between different NK cell functional markers (as seen in Supplemental Fig. 2D–I) are also seen when NK cells are stimulated with high concentrations of exogenous IL-12 and IL-18 (data not shown). Stimulation of purified NK cells with H3N2 alone or with IL-15 induced no significant activation, confirming an accessory cell requirement for the virus-induced response and IL-15–mediated enhancement (IFN-γ percentage at 18 h: H3N2 median 0.29%, IQR 0.25–0.368%, H3N2+IL-15 median 0.465%, IQR 0.308–0.84; CD107a percentage at 18 h: H3N2 median 0.93%, IQR 0.605–1.293%, H3N2+IL-15 median 1.68%, IQR 0.75–2.475%).

### IL-15 enhances IL-12 production by mDCs

**FIGURE 5.**
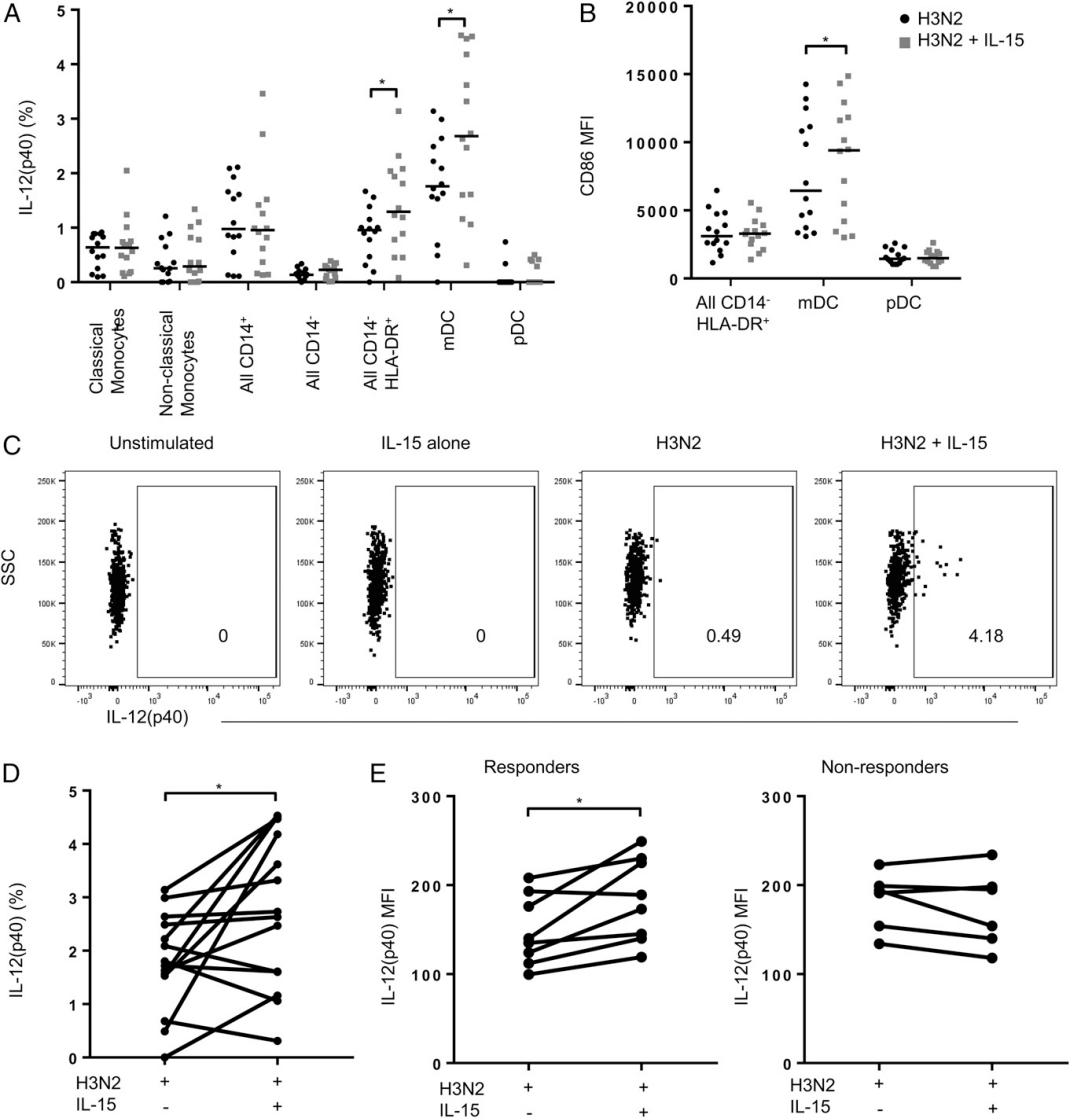
IL-15 enhances IL-12 production by mDCs. PBMCs were cultured with H3N2 or H3N2 with IL-15 (*n* = 14) for 18 h, and cells were stained for DC and monocyte phenotypic markers and intracellular IL-12. Single live cells were further gated as lineage (CD3, CD19, CD56) negative, classical (CD14^+^CD16^−^), and nonclassical (CD14^−^CD16^+^) monocytes, all CD14^+^ and CD14^−^ cells, all CD14^−^HLA-DR^+^ DCs, mDCs (CD123^−^CD11c^+^), and pDCs (CD123^+^CD11c^−^). IL-12^+^ events (**A**) and CD86 MFI (**B**) for each cell type was gated using fluorescence minus one controls and shown as one data point per donor; the horizontal line represents the median. (**C**) Flow cytometry gating strategy for IL-12^+^ mDCs is shown in one representative individual; numbers denote the percentage of IL-12^+^ mDCs. Before-and-after plots show the percentage of mDCs expressing IL-12 for each individual when stimulated with H3N2 ^+/−^ IL-15 (**D**) and the corresponding IL-12 MFI for each responder and nonresponder with IL-15 costimulation (**E**). *p < 0.05, Wilcoxon signed-rank test.

To determine the source of IL-15–induced IL-12 within PBMCs, stimulations were performed with H3N2 in the presence or absence of IL-15 for 18 h, and cells were stained for DC/monocyte phenotypic markers and for intracellular IL-12(p40). DC populations were gated as lineage^−^CD14^−^HLA-DR^+^ cells and further split into CD123^−^CD11c^+^ mDC and CD123^+^CD11c^−^ plasmacytoid DC (pDC) populations. The majority of IL-12–producing cells were mDCs rather than pDCs, classical (CD14^+^CD16^−^) monocytes, or nonclassical (CD14^−^CD16^+^) monocytes (Fig. 5A). Up to 3.1% of mDCs expressed IL-12 in response to H3N2, and a significantly higher proportion (4.5%) were IL-12^+^ with the combination of H3N2 and IL-15 (a median 1.9-fold increase) (Fig. 5A). Furthermore, IL-15–mediated enhancement of IL-12 production was observed only in DCs and was associated with increased expression (MFI) of CD86 (Fig. 5B). Little to no IL-12 was detected in unstimulated mDCs or in those treated with IL-15 alone (Fig. 5C). Of the 14 individuals tested, 8 (57.1%) showed an increase in IL-12 production by mDCs with H3N2 plus IL-15 compared with H3N2 alone, corresponding with the proportion of responders determined by Luminex detection of IL-12 in supernatant (Fig. 5D). Among these eight responders, there was also an increase in the MFI of IL-12 staining of mDCs cultured with H3N2 plus IL-15 compared with H3N2 alone (Fig. 5E). These data suggest that IL-15 potentiates NK cell responses to H3N2 by enhancing maturation (CD86 expression) and IL-12 production specifically from mDCs.

### Enhancement in NK cell function by IL-15 is observed in HCMV-seropositive and -seronegative individuals

**FIGURE 6.**
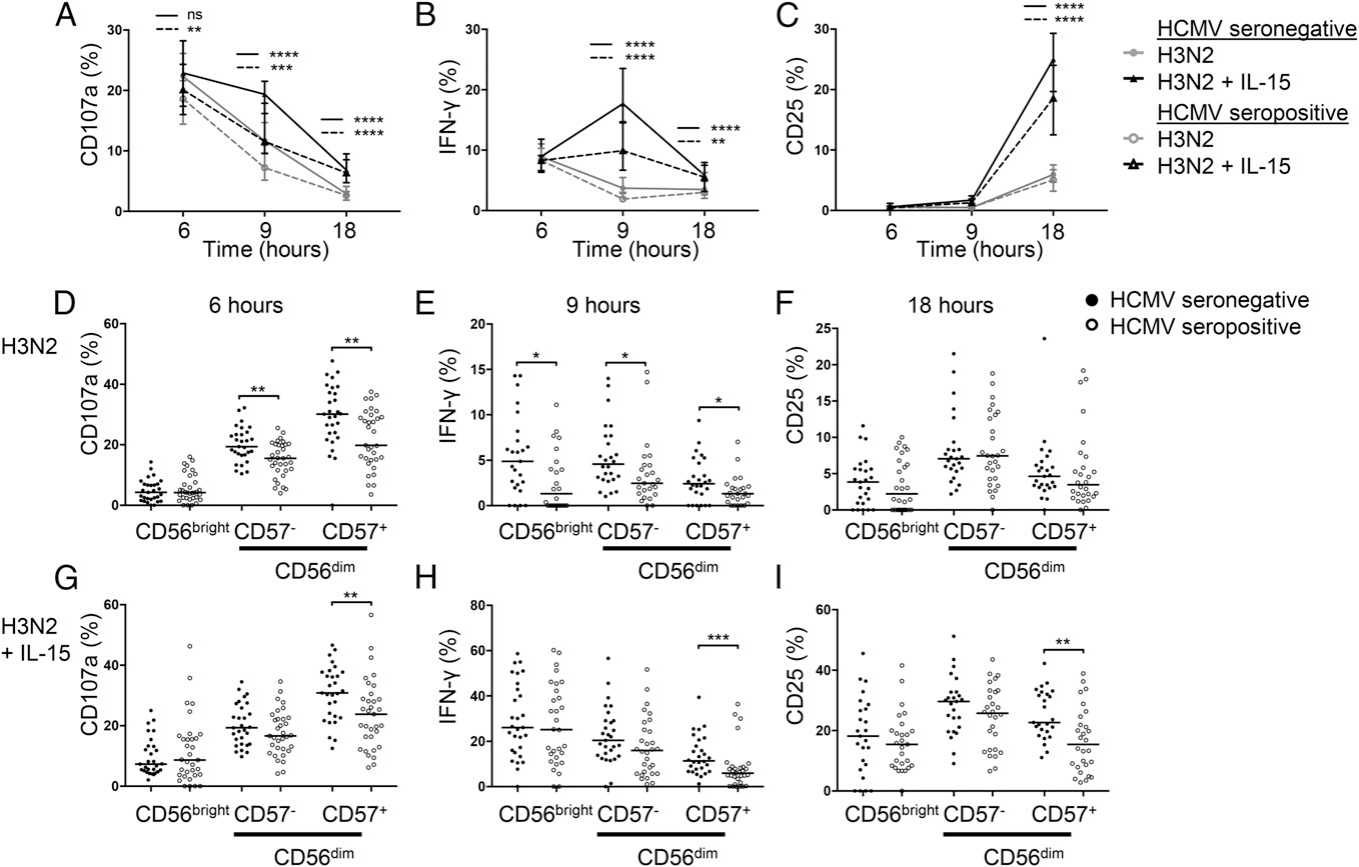
Enhancement in NK cell function by IL-15 is observed in HCMV-seropositive and -seronegative individuals. HCMV serostatus for each individual was determined (*n* = 35 seropositive and *n* = 38 seronegative). CD107a (**A**), IFN-γ (**B**), and CD25 (**C**) expression within each group after stimulation with H3N2 or H3N2 plus IL-15 for 6, 9, or 18 h. NK cell responses attributed to each differentiation subset determined by the expression of CD56 and CD57, CD56^bright^, CD56^dim^CD57^−^, and CD56^dim^CD57^+^ were analyzed (flow cytometry gating strategy in Supplemental Fig. 1A). Graphs show median with 95% confidence interval. CD107a (**D** and **G**), IFN-γ (**E** and **H**), and CD25 (**F** and **I**) expression within each subset is shown for HCMV-seropositive and -seronegative individuals; only the peak time point for each response is shown. Each dot represents an individual donor; the horizontal line represents the median. *p < 0.05, **p < 0.01, ***p < 0.001, ****p < 0.0001, H3N2 versus H3N2 plus IL-15, Wilcoxon signed-rank test (A–C), unpaired Mann–Whitney *U* test (D–I). ns, not significant.

We (19, 20), and other investigators (21, 22) have reported altered NK cell functions in HCMV-seropositive individuals. HCMV-seropositive individuals also respond less well to exogenous cytokines and vaccine Ags (including H3N2), and this is only partially explained by accelerated NK cell differentiation (19, 20). We again observed lower NK cell CD107a, IFN-γ, and CD25 responses to H3N2 among HCMV-seropositive donors than among HCMV-seronegative donors (Fig. 6). Low concentrations of IL-15 enhanced the responses of HCMV-seropositive and -seronegative subjects but could not fully restore the NK cell response of HCMV-seropositive subjects to the level seen in HCMV-seronegative subjects (Fig. 6A–C). Interestingly, however, IL-15 completely restored the IFN-γ response of CD56^bright^ and CD56^dim^CD57^−^ NK cells, but not CD56^dim^CD57^+^ cells, in HCMV-seropositive individuals (Fig. 6E, 6H). This suggests that IL-15 preferentially affects immature NK cells, normalizing their IFN-γ response to H3N2 to the level seen in HCMV-seronegative individuals. With the exception of a reduced IFN-γ response, which may be accounted for by reduced production from NK cells in seropositive individuals, there was no difference in the in vitro production of lymphoid or myeloid cell–derived cytokines in response to H3N2 between HCMV-seropositive and -seronegative donors, irrespective of the presence or absence of IL-15 (Supplemental Fig. 3).

## Discussion

Many studies of the effect of IL-15 on NK cell activation focus on IL-15 *trans*-presentation or direct activation of NK cells with high concentrations of IL-15 (typically between 5 and 50 ng/ml) in combination with other cytokines (5, 10). Moreover, the synergy between cytokines and pathogen-derived signals for NK cell activation has typically been studied only for high concentrations of cytokines acting on isolated NK cells, precluding consideration of the potential indirect effects of the pathogen, the cytokines, or both. To characterize more deeply the potential for synergy between IL-15 and pathogen-derived signals in NK cell activation, we have conducted a comprehensive analysis of NK cell CD107a, IFN-γ, and CD25 expression and myeloid cell–derived cytokine secretion in response to H3N2 in the presence or absence of an extremely low concentration of IL-15.

Low concentrations of IL-15 enhanced the innate cytokine response to influenza virus, and this increased cytokine (primarily IL-12) was associated with potentiation of NK cell function. Importantly, the concentrations of endogenous IL-12 induced by this synergistic interaction between influenza virus and IL-15 are of the same order of magnitude as the lowest concentrations of IL-12 that we have previously shown to effectively synergize with IL-15 and other common γ-chain family cytokines for NK cell activation in vitro (13). The measured concentration of IL-12 and the concentration of IL-15 used in this study are ≥5-fold lower than the described effective concentrations for NK cell activation with single cytokines and may be more physiologically relevant than the higher concentrations previously studied in vitro. The low in vitro concentration used in this study is within the range for the maximal serum concentration achieved therapeutically in patients with metastatic malignant melanoma or renal cancer after a low-dose transfusion of 0.3 μg/kg/day recombinant human IL-15 and which resulted in increases in innate cytokines (23).

In this article, we have shown that IL-15 preferentially enhances virus-induced mDC maturation (measured by upregulation of costimulatory marker CD86), as well as cytokine secretion, compared with other DC subsets and monocytes and that, together, this heightens and sustains NK cell activation. This role for mDCs is fully in line with the known pathogen-recognition repertoire and cytokine-production profile of mDCs (24, 25). Similarly, the essential role for DC-derived IL-12 in NK cell activation is in line with published data, including data showing that IL-12 synergizes with specific Ab for NK cell–mediated Ab-dependent cell–mediated cytotoxicity of tumors (26, 27), that NK cell activation by HPV-like particle–matured DCs is reversed by IL-12–blocking Abs (28), and that exogenous IL-12 can restore NK cell function in HIV-exposed, but uninfected, infants (29). Interestingly, IL-15 synergizes well in vitro with IL-12 and IL-18 but not with other IL-2Rγ-chain–dependent cytokines, including IL-2 and IL-21, suggesting some redundancy between the latter pathways (13). This is also consistent with our observation that neutralization of endogenous IL-2 did not reduce NK cell IFN-γ in response to H3N2 and IL-15.

Enhancement of NK cell function by IL-15 is well established (30). IL-15 has also been reported to enhance DC maturation (measured by upregulation of CD40, CD86, and MHC class II expression) in mice (17, 31). In line with our observations in humans, DCs cultured with HPV-like particle matured more efficiently in the presence of IL-15, and this correlated with enhanced NK cell activation and killing of HPV-infected tumor cells (17). However, the role of IL-15 enhancement of IL-12 production by peripheral DCs has not been previously appreciated or linked to enhanced NK cell responses. Interestingly, one study demonstrated that IL-15 enhancement of IL-12 secretion by a PMA-activated U937 monocytic cell line was associated with increased ability to kill intracellular *Leishmania* parasites, suggestive of a potential role for such mechanisms in protection against infection (32).

The cytotoxic and cytokine-producing roles of NK cells have traditionally been ascribed to distinct NK cell subsets (CD56^dim^ and CD56^bright^, respectively), and this dichotomy has rather obscured the potential for NK cells to perform both functions simultaneously (i.e., to be polyfunctional). Nevertheless, polyfunctional CD56^dim^CD62L^+^ NK cells (with dual ability to produce IFN-γ and become cytotoxic) can be induced by stimulation with high concentrations (50 ng/ml) of IL-12 and IL-18 (33), and TLR stimulation induced polyfunctional NK cells (expressing two or more of CD107a, IFN-γ, and TNF-α) in HIV-1–exposed seronegative, but not seropositive, individuals (34). We defined polyfunctionality as simultaneous expression of two or more of IFN-γ, CD107a, and CD25 by any NK cell subset; no polyfunctional cells were induced by IL-15 alone, and very few were detected in response to influenza virus alone. However, stimulation with IL-15 and virus induced significant numbers of double- and triple-positive NK cells, suggesting that synergy (likely at the level of mDCs) between IL-15 and pattern recognition receptor signaling may be necessary for the induction of polyfunctional NK cells. This may be due, in part, to the dependence of NK cells on IL-12 to drive IFN-γ production and on IFN-α and IL-15 to drive cytotoxicity (2, 35).

Our observation that IL-15 restored the impaired responses of HCMV-seropositive individuals only among the less differentiated CD56^bright^ and CD56^dim^CD57^−^ NK cell subsets, as well as that (with the exception of IFN-γ production) there were no differences in cytokine production by PBMCs from HCMV-seropositive and -seronegative donors, suggests that polyfunctionality may arise from broadening the effector function of less mature NK subsets rather than any effect on mature NK subsets. This effect is entirely consistent with IL-12–mediated IL-15 enhancement of NK cell responses, IL-12R expression, and, therefore, responsiveness being progressively lost during NK cell differentiation and in adaptive NK cell subsets (18, 36). Moreover, these data suggest that reduced NK cell responses in HCMV-seropositive individuals are not due to an accessory cell defect but may result from intrinsic changes in more-differentiated NK cells (18, 22). This hypothesis, that IL-15 broadens the functional response of immature NK cells, is further supported by evidence that degranulation and IFN-γ and TNF-α production from CD56^bright^ NK cells are potently enhanced by exposure to multiple myeloma or acute myeloid leukemia target cells after in vivo therapy with the IL-15R agonist ALT803 (37). The use of an IL-15/IL-15Rα superagonist may further boost NK cell responses to H3N2; however, this would be expected to act on NK cells directly, because IL-15 is already complexed to its receptor, therefore potentially bypassing accessory cell–dependent effects.

In summary, we have revealed an unexpected impact of very low concentrations of IL-15 on the production of cytokines, in particular IL-12 from mDCs, which, in turn, plays a vital role in boosting NK cell responses to influenza virus (summarized in Supplemental Fig. 4). In addition to increasing the overall frequencies of responding NK cells, IL-15 promotes the generation of polyfunctional NK cells. These studies suggest that the use of very low dose IL-15 may be a strategy for enhancing and broadening NK cell effector function in immunotherapy and in enhancing vaccine responses.

## Supplementary Material

Data Supplement
