# Supplementary material for: IL-15 Promotes Polyfunctional NK Cell Responses to Influenza by Boosting IL-12 Production
Source: J Immunol. 2018 Feb 28;200(8):2738–47. doi: 10.4049/jimmunol.1701614 (PMC5890538; doi:10.4049/jimmunol.1701614)
Supplement: Data Supplement [file JI_1701614.zip › JI_1701614_Supplemental_Figures_1.pdf]

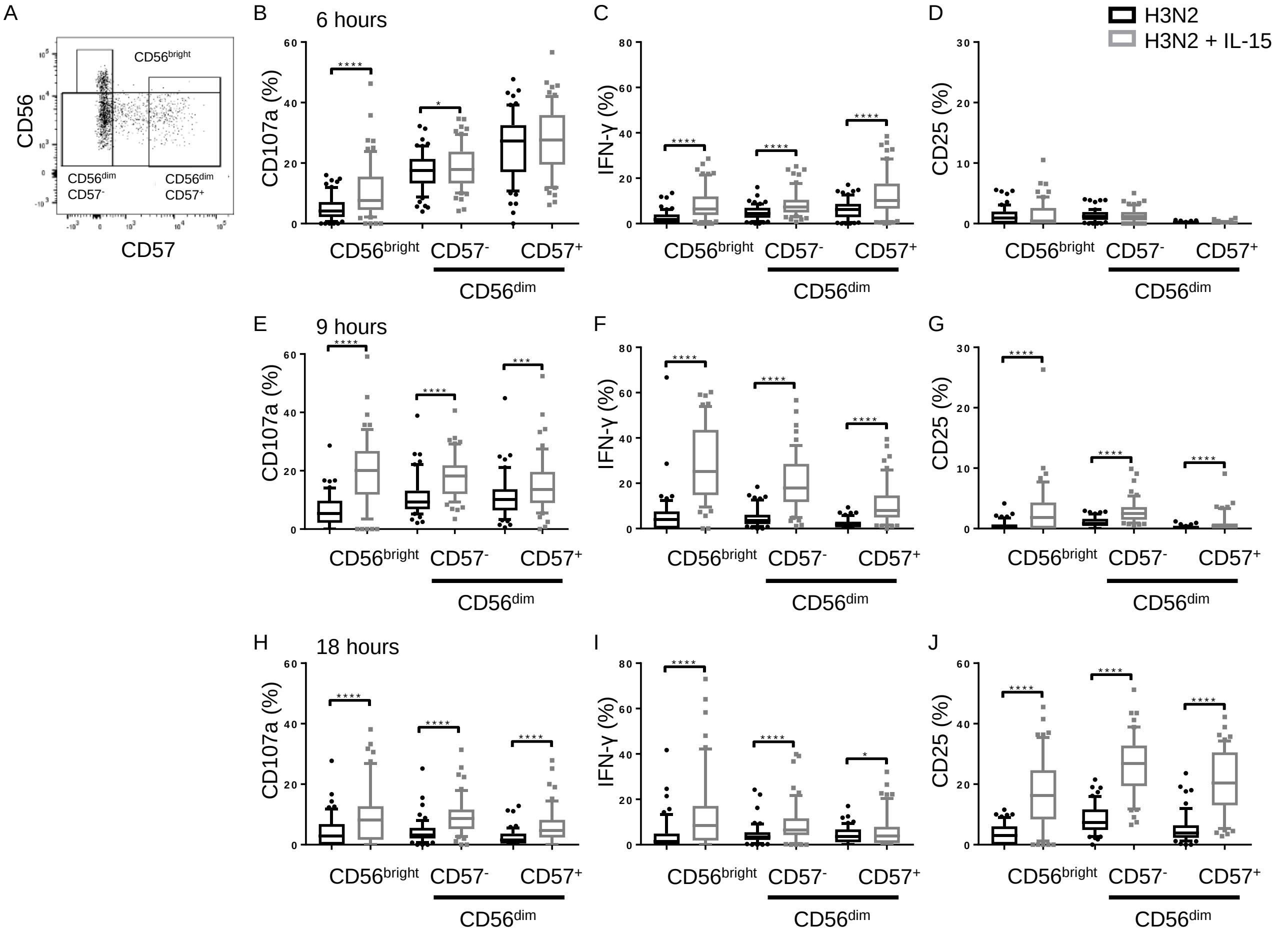

Supplementary figure 1; related to Figure 1. IL-15 boosts NK cell functional responses to H3N2 vaccine antigen in all NK cell differentiation subsets.

NK cell differentiation subsets are determined by the expression of CD56 and CD57 into CD56^{bright}, CD56^{dim}CD57^{-} CD56^{dim}CD57^{+} subsets (Gating strategy in A). CD107a (B, E, H), IFN-γ (C, F, I) and CD25 (D, G, J) expression within these subsets was determined after stimulation with H3N2 (black) and H3N2 plus IL-15 (grey) for6 (B-D), 9 (E-G) and 18 hours (H-J) p, (n=62). Graphs are box and whisker plots with 10-90th percentile and paired statistical analysis between with and without IL-15 by Wilcoxon signed-rank test. *p, 0.05, **p, 0.01, ***p, 0.001, ****p, 0.0001.

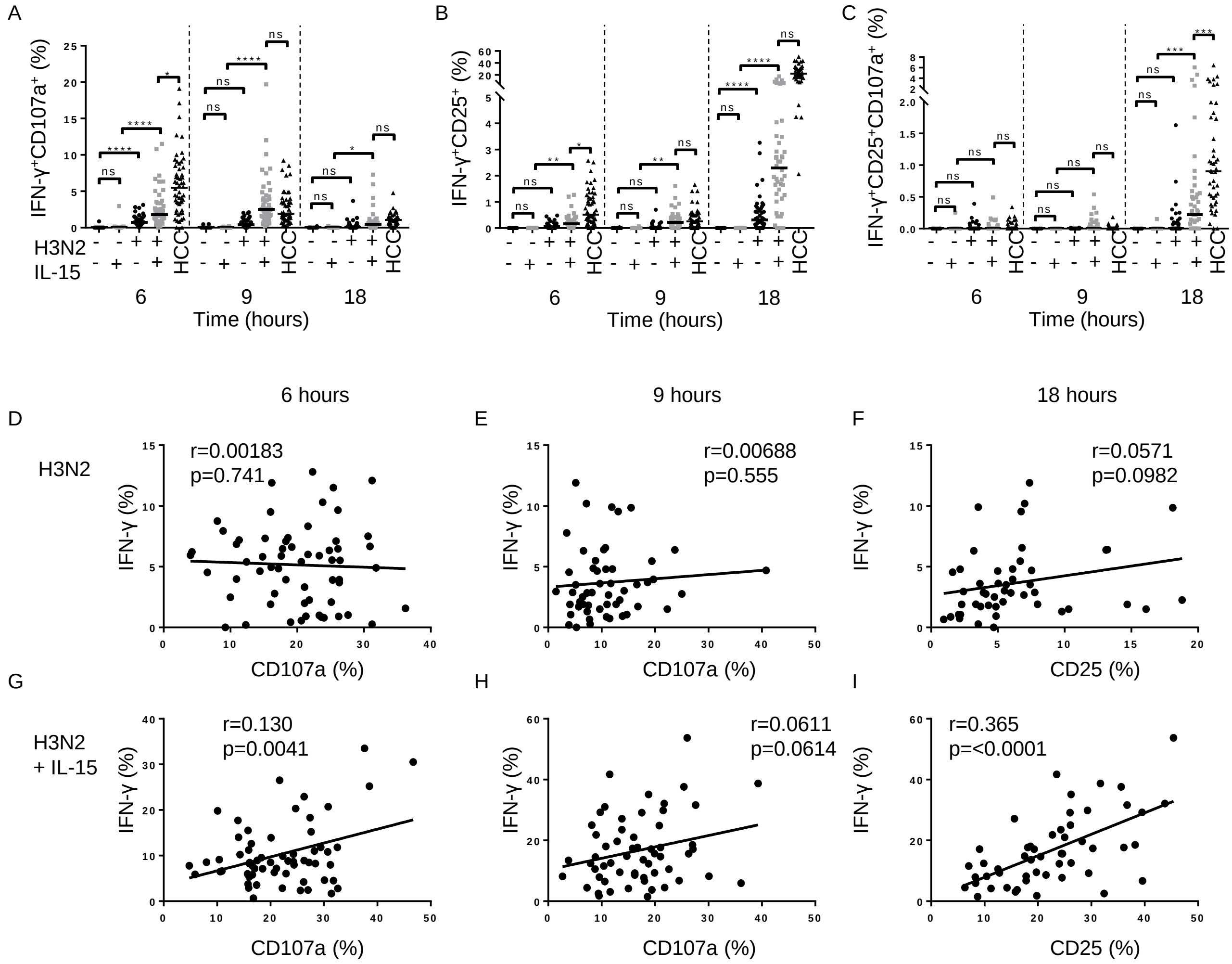

Supplementary figure 2; related to Figure 2. No detection of polyfunctional NK cells in cultures containing only IL-15, the relationship between NK cell functional responses becomes more positively correlated with the presence of IL-15.

Frequencies of IFN-γ^+CD107a$^+$ (A), IFN-γ^+CD25$^+$ (B) double positive and IFN-γ^+CD25$^+$CD107a$^+$ (C) triple positive NK cells after H3N2 stimulation with and without IL-15 or with high concentration cytokines (HCC) consisting of IL-12 and IL-18 (5ng/ml and 50ng/ml respectively) are shown for each time point (unstimulated and IL-15 alone; n=12, H3N2, H3N2 plus IL-15 and HCC; n=62). (D-I) Graphs shown the correlation between NK cell IFN-γ production and CD107a or CD25 expression after 6 (D, G), 9 (E, H) or 18 (F, I) hours stimulation with H3N2 (D-F) or H3N2 with IL-15 (G-I) (n=62). Graphs show one data point per donor with the line at median, statistical analysis by one way ANOVA Kruskal-Wallis test with Dunns multiple comparisons. ns, not significant, *p, 0.05, **p, 0.01, ***p, 0.001, ****p, 0.0001. Correlations were measured by linear regression, statistical significance determined as a p value of below 0.05.

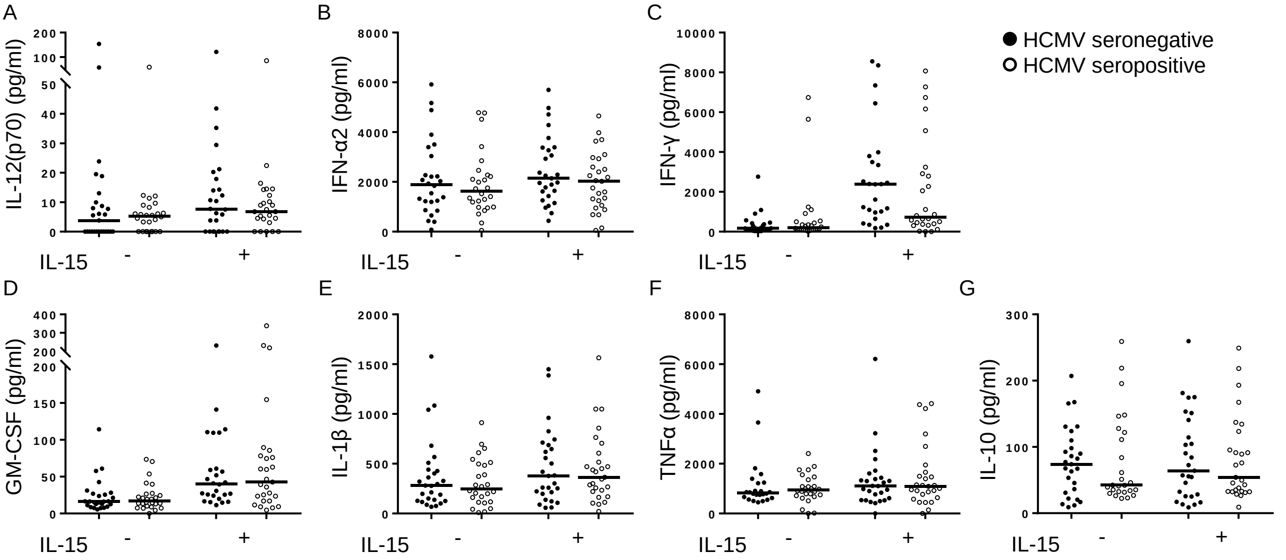

Supplementary Figure 3; related to figure 6. Cytokine release is not significantly different between HCMV seropositive and seronegative groups.

Concentrations of IL-12p70 (A), IFN-α2 (B), IFN-γ (C), GM-CSF (D), IL-1β (E), TNF-α (F) and IL-10 (G) in supernatants after 18 hours stimulation were determined by Luminex technology and split in to HCMV seropositive and seronegative groups (n=35 seropositive; open symbols, n=38 seronegative; closed symbols). Graphs show one dot per donor with line at median, paired statistical analysis between H3N2 alone and H3N2 with IL-15 by Wilcoxon signed-rank test. *p, 0.05, **p, 0.01, ***p, 0.001.

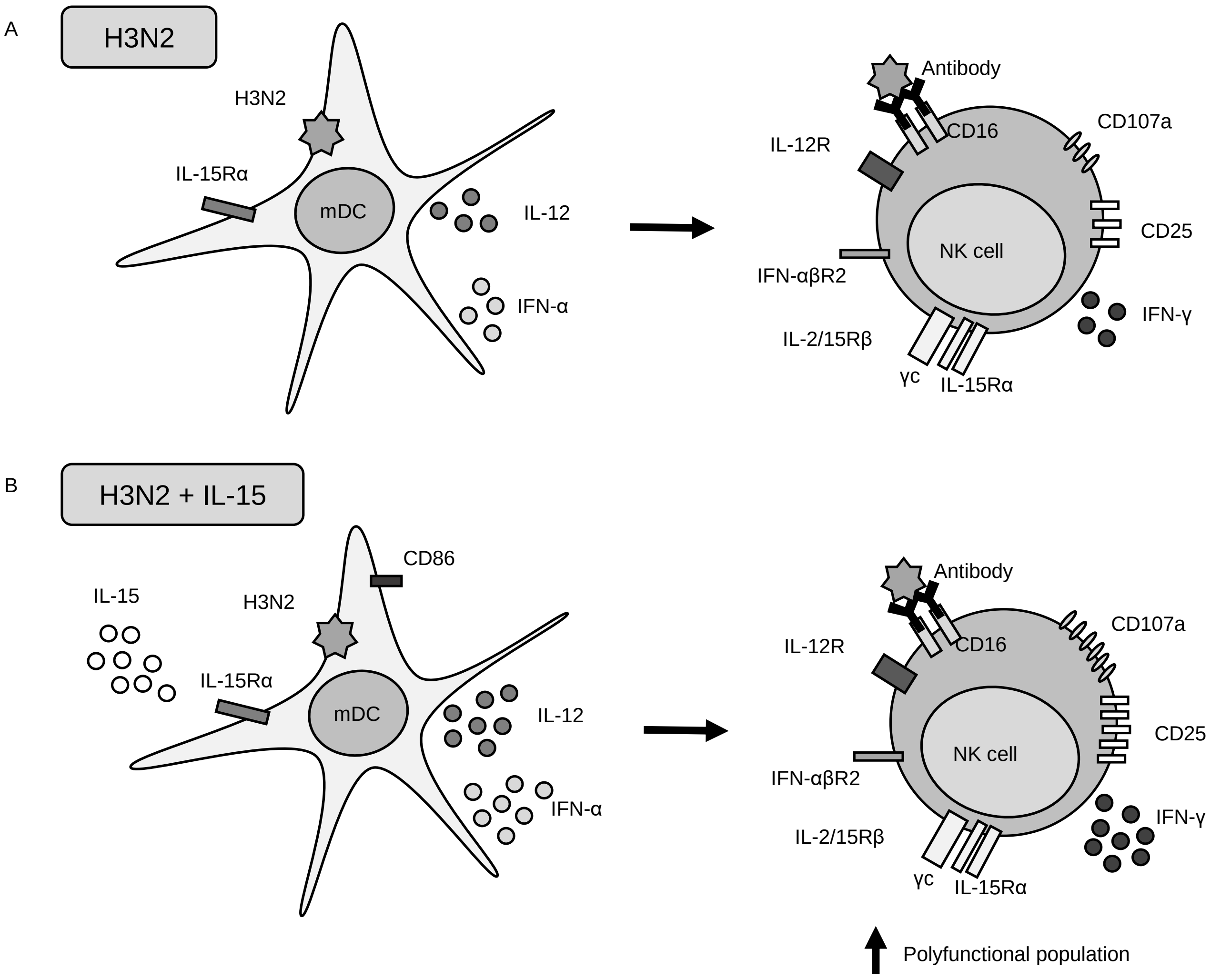

Supplementary Figure 4. A schematic representation of the effect of IL-15 on NK cell responses to H3N2.

H3N2 induced IL-12 secretion from mDCs is enhanced in the presence of very low concentrations of IL-15, leading to enhanced NK cell responses and higher frequencies of polyfunctional NK cells independently of trans-presentation or direct NK cell IL-15R binding.
